# Supplementary material for: Lesion of striatal patches disrupts habitual behaviors and increases behavioral variability
Source: PLoS One. 2020 Jan 8;15(1):e0224715. doi: 10.1371/journal.pone.0224715 (PMC6948820; doi:10.1371/journal.pone.0224715)
Supplement: Attachment — Submitted filename: Response to Reviewers.docx [file pone.0224715.s003.docx]

Jeff A Beeler, Ph.D.

Academic Editor

*PLOS ONE*

 December, 18 2019

 ATT: Manuscript Re-submission to *PLOS ONE* (PONE-D-19-28957R1)

Dear Dr. Beeler,

We would like to again thank you and the reviewers for insightful comments and prompt processing of our manuscript. Below we address each comment raised by the reviewers. We have created two new panels in Figure 4, and have updated our Graphpad Prism file accordingly. Moreover, we have included new discussion and edited wording in several places in the document. Again, we feel the work has been made stronger through this process. Below you will find our responses to each concern with reviewer comments italicized, changes to text in bold, and our own comments in plain text.

*Reviewer #1: My concerns have been addressed by the authors. the manuscript has been improved considerably. The discussion now includes better acknowledgment of previous work, including the difference between omission and devaluation as assays for habitual performance. The additional discussion of different aspects of habitual behavior is also appropriate. I think the paper is now publishable.*

We again thank the reviewer for their constructive feedback on this work, we agree that the manuscript has benefited significantly from the inclusion of these topics.

*Reviewer #2: Overall, the authors have done a good job at addressing my previous comments. My additional comments are listed below.*

*- Regarding my primary concern about the devaluation procedure, the authors claim that “the current approach using home-cage chow and liquid sucrose has been successfully employed in the literature (He et al. 2016; Li et al. 2018)”. The results of the He et al. paper alleviate my concern because the mice are food-deprived and when they are given selective satiety tests after training on a random ratio schedule, they show instrumental sensitivity to devaluation. This is encouraging. The Li et al. paper is less relevant because the mice are not reported as food-deprived, so I recommend removing this citation.*

This citation has been removed. *The authors also appeal to the fact that other researches have used a combination of liquid and solid reinforcers during devaluation tests, but this does not relate to my concern and distracts from the main issue. My concern is a higher rate of consumption during devalued vs. valued tests (I would be concerned about this even if the authors used wildtype mice, so translating this concern into a caveat should generalize beyond Sepw1 mice). If the assignment of solid and liquid reinforcers is balanced across subjects, this concern goes away (mostly). I strongly recommend the authors rephrase the following paragraph by removing the first sentence and amending the second sentence to mean mice in general (not just Sepw1 mice):*

*“The combination of liquid and solid reinforcers used across devaluation and valuation trials is common (Gremel and Costa 2013; Gremel et al. 2016; He et al. 2016; Li et al. 2018; Renteria et al. 2018). Nevertheless, the lack difference in our devaluation and valuation trials could be attributed to this asymmetry in reward, which Sepw1 mice may be particularly sensitive to”.*

We apologize for the confusion on this point. This has been done. Lines 458-465 now read:

**“First, the use of home-cage chow and liquid sucrose rewards could represent an asymmetrical manipulation between devaluation and valuation probe trials, which may have impacted the results of these probe trials. However, the approach used here has been utilized in a previous study, and these mice remained sensitive to devaluation [44]. Nevertheless, the lack of difference in our probes could be attributed to potential asymmetry in consumption before devaluation and valuation probes.”**

*- I previously recommended that the authors display and analyze the raw pressing rates from the selective satiety tests. However, in the revised paper the authors have only presented and analyzed raw pressing rates from the devalued condition but not the valued condition. I apologize if I was not clear, but this is the standard way of analyzing devaluation test data (comparing valued vs. devalued for each group). Without the raw pressing rates from the valued condition, there is no way to compare figures 4A and 4C. If the authors are going to present the raw pressing rates from the devalued condition, they must also present the raw pressing rates from the valued condition.*

We apologize for the confusion here as well. We have now performed this analysis and included two new panels in Figure 4. As shown below, presses between devaluation and valuation trials were not different in either control or lesioned groups (A-B). This result has been updated in Results (Line 305-307), Figure legend 4 (Line 321-322), and the paired student’s t-test used to compare these is now in Statistical Analysis (Line 194-195).

*Reviewer #3: The authors have largley addressed my concerns. The main one being the leakiness of the line. It still feels a bit misleading to state investigation of a patch deletion on these behaviors. The question of patch like-due to development is interesting, but a separate investigation.*

The author highlights an important limitation of the current work. While the Sepw1 line has been used in many other studies to determine patch function (Crittenden *et al.* 2016; Smith *et al.* 2016; Yoshizawa *et al.* 2018), the off-site targeting of exopatch neurons remains problematic. Other Cre lines are available and provide viable alternatives, including Mash1-CreER (Bloem *et al.* 2017), 599-CreER (McGregor *et al.* 2019), or Oprm1-Cre (Märtin *et al.* 2019), though each of these lines suffers from some level of exopatch Cre expression (or expression of Cre in matrix). In acknowledgment of this shortcoming, we have added the following paragraph to discussion:

“**While Sepw1 NP67 mice have preferential expression of Cre recombinase in patch projection neurons, a limitation of the current work is the expression of Cre in ‘exo-patch’ neurons (Smith et al 2017), resulting in the lesioning of both patch and exo-patch neurons (Figure 1). Exo-patch neurons have similar gene expression and connectivity profiles to patch neurons (Smith et al 2017), but they fall outside of traditionally defined patches (Crittenden et al 2011). The Sepw1 NP67 line has been previously used to study patch connectivity (Smith et al, Crittenden et al 2016) and activity (Yoshizawa et al 2018). Other recent studies have utilized alternative Cre lines to target patches, including Mash1-CreER (Bloem et al 2017), 599-CreER (McGregor et al 2019), or Oprm1-Cre (Märtin et al 2019), though each of these lines also has some off-target labeling of exo-patch or matrix neurons. Thus, while the current work suggests lesions preferentially targeting patches impair aspects of habitual behavior, it remains unknown what role exo-patch neurons play in this process**.”

References

Bloem B., Huda R., Sur M. and Graybiel A. M. (2017) Two-photon imaging in mice shows striosomes and matrix have overlapping but differential reinforcement-related responses. Elife. 6, 10.7554/eLife.32353.

Crittenden J. R., Tillberg P. W., Riad M. H., Shima Y., Gerfen C. R., Curry J., Housman D. E., Nelson S. B., Boyden E. S. and Graybiel A. M. (2016) Striosome-dendron bouquets highlight a unique striatonigral circuit targeting dopamine-containing neurons. Proc. Natl. Acad. Sci. U. S. A. 113, 11318-11323.

Märtin A., Calvigioni D., Tzortzi O., Fuzik J., Wärnberg E. and Meletis K. (2019) A Spatiomolecular Map of the Striatum. bioRxiv, 613596. (Preprint)

McGregor M. M., McKinsey G. L., Girasole A. E., Bair-Marshall C. J., Rubenstein J. L. R. and Nelson A. B. (2019) Functionally Distinct Connectivity of Developmentally Targeted Striosome Neurons. Cell. Rep. 29, 1419-1428.e5.

Smith J. B., Klug J. R., Ross D. L., Howard C. D., Hollon N. G., Ko V. I., Hoffman H., Callaway E. M., Gerfen C. R. and Jin X. (2016) Genetic-Based Dissection Unveils the Inputs and Outputs of Striatal Patch and Matrix Compartments. Neuron. 91, 1069-1084.

Yoshizawa T., Ito M. and Doya K. (2018) Reward-Predictive Neural Activities in Striatal Striosome Compartments. eNeuro. 5, 10.1523/ENEURO.0367-Feb.

 Again, we thank you and the reviewers for careful consideration of our work. We look forward to re-review of our manuscript.

We would be grateful to be considered in the Neuroscience of Reward and Decision Making Call for Papers and we have no changes to our financial disclosure.

 Sincerely,


 Christopher D. Howard, Ph.D.
 Assistant Professor of Neuroscience
 Department of Neuroscience
 Oberlin College; Oberlin, OH 44074
 [choward@oberlin.edu](mailto:choward@oberlin.edu); (440)-775-8015
